# Reliability Study of Wood–Plastic Composites Based on Probabilistic Finite Elements

**DOI:** 10.3390/polym15020312

**Published:** 2023-01-07

**Authors:** Li Feng, Dejin Wang, Jun Yan

**Affiliations:** College of Mechanical and Electrical Engineering, Northeast Forestry University, Harbin 150040, China

**Keywords:** WPC, reliability, finite element analysis

## Abstract

In order to further expand the application field of wood–plastic composites, it is necessary to study the reliability of this material in practical applications. Therefore, this work takes the maximum stress theory as the failure criterion and uses the finite element method to simulate the reliability of the WPC specimen. Based on the simulation results, the relationship between reliability and random variables such as geometric parameters and external load is analyzed. Finite element simulations are carried out for each group of specimens under the same operating environment to analyze the influence of process parameters such as the wood flour content, granulation temperature, coupling agent content and screw speed on the reliability of the specimens during the manufacturing process. The results show that the wood flour content has the greatest influence on the reliability of the specimens when the wood–plastic composites are used as building paving materials, followed by the granulation temperature, coupling agent content and screw speed, which provides a basis for the selection of the manufacturing process parameters of WPC based on reliability.

## 1. Introduction

Wood–plastic composites are a composite material with good physical properties, replacing the usual resin binder with polyethylene, polypropylene, polyvinyl chloride, etc., and mixing it with wood cellulose or other types of plant cellulose [1,2]. Since the cellulose in the wood–plastic composite is fully mixed with plastic, they have high compressive and bending resistance and exhibit relatively low deformation over time under continuous loads [3,4]. The durability is significantly better than that of ordinary wood, and they have shown potential as structural materials. Currently, wood–plastic composites are mainly used in the construction industry for paving panels and railing structures [5,6,7,8]. These structures not only carry various static loads, but also may be subjected to various random loads in vertical or horizontal directions. Therefore, it is of great significance to study the reliability of wood–plastic composites under actual use and to design the structural dimensions and processing parameters according to the magnitude of the loads that they are subjected to.

The reliability of wood–plastic composites has been studied by several scholars. The reliability of laminated wood plastic lumber was analyzed by the Monte Carlo method [9]; the fatigue characteristics and reliability of wood flour-filled polypropylene (PP) composites were investigated, and a probabilistic method for assessing fatigue reliability was proposed [10]; the reliability of wood–plastic composite laminates under transverse loads was analyzed based on the Monte Carlo method, β-method and first-order second-order moment method [11]; the failure probability and reliability of single-span double-layered wood–plastic frame structures were evaluated using the primary second-order moment method and the modified primary second-order moment method, respectively, and the reliability of wood–plastic composite panels of different densities when used as ammunition boxes was analyzed [12,13]. A reliability study was conducted on a wood–plastic composite hull, and the limit state equation of the composite-reinforced panel material was proposed [14,15]; the effects of the coupling agent, test frequency and stress ratio on the fatigue life and reliability of wood–plastic composite panels used in footbridges were investigated experimentally [16]. The surface properties and structural strength of colored wood–plastic composites were studied under the corrosive environment of seawater, and the relationship between the coloring pigments and the reliability of the wood–plastic composites was analyzed [17,18,19]. The fatigue analysis and reliability of polypropylene (PP)/wood flour (WF) composites were investigated, and it was suggested that the fatigue life obtained from the lower 95% confidence limit can be used as a material reliability index for the safe fatigue design of composites [20,21]. Since wood–plastic composites are multi-phase, non-homogeneous, anisotropic materials, their mechanical properties, characteristics and damage forms are very much related to the fiber direction of the material. The microscopic mechanical property theory is too computationally demanding, so it is very difficult to apply to analyze the reliability of wood–plastic composites. In many cases, the problem still needs to be studied empirically by experimentation, and it is difficult to give an accurate assessment of its reliability from a theoretical point of view. At present, most of the studies on the reliability of wood–plastic composites use the first second-order moment method for analysis, but this method requires the assumption of some variables and the addition of some coefficients. The accuracy of the analysis results will have some deviation due to the introduction of the coefficients. Therefore, the results obtained are basically approximate solutions under certain assumptions.

## 2. Materials and Methods

The materials for this test were wood flour (20–40 mesh) and the high-density polyethylene of type 5000S produced by Petroleum Petrochemical Company, Daqing, China. The coupling agent was maleic anhydride-grafted polyethylene produced by Baichen Co, Guangzhou, China. After selecting the material, the wood flour was first dried to a moisture content of less than 3%. After drying the wood flour, the wood flour, polyethylene and the coupling agent after grinding were weighed using an electronic analytical balance with accuracy of 0.1 mg. In order to facilitate the subsequent analysis of reliability in relation to the process parameters, the test was designed with the wood flour content, granulation temperature, coupling agent content and screw speed as process variables during the manufacture of the wood–plastic composite. The specimens were divided into 20 groups of five specimens each. The processing parameters of each group of specimens are shown in Table 1. Each group of five was subjected to a test, and each group determined the step size of the variable based on the range of the variable, e.g., for groups 1 to 5, the wood flour content was 30%, 40%, 50%, 60% and 70%, respectively, with other parameters remaining the same and other groups similar.

## 3. Mechanical Property Test

**Figure 1:**
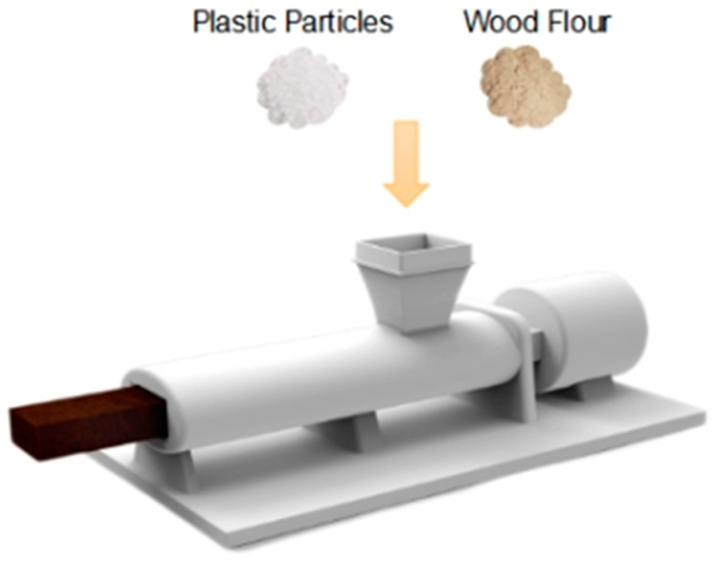
Process diagram of extrusion molding method.

After determining the proportions of the components, each group was placed together and numbered for ease of handling. After the numbering was completed, the component materials were placed in the SHR-A high-speed mixing mixer by Mingrunda Technology Co., Suzhou, China, according to the numbering order, so that the wood flour, polyethylene and the coupling agent were fully mixed. After mixing, the material was granulated in the SJSH30 intermeshing co-rotating twin screw extruder by Horizon Technology Co., Guanzhou, China. The granulation temperature and screw speed were adjusted according to Table 1 as the numbering changed. After granulation, the material was poured into the SJ45 single screw extruder by Flanders Machinery Co., Zhangjiagang, China, for the extrusion of the profile, as shown in Figure 1. During the extrusion of the profiles, it was important to check for changes in the material of the profiles. The extruded profiles were numbered and sorted according to the previous numbers.

**Figure 2:**
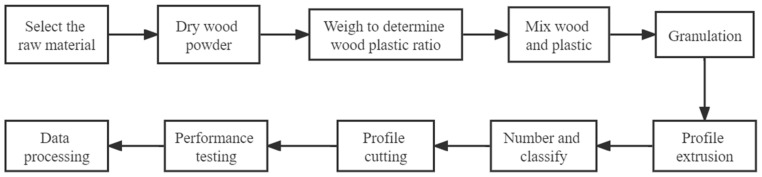
Main process of the test.

After all the profiles had been extruded, the profiles were cut according to the geometry of the specimens, and each of the specimens obtained after cutting was numbered to facilitate subsequent operations. The geometry of the specimens was 164 mm × 40 mm × 4 mm, and each group of specimens consisted of 5 plates. After the profile was cut, the resulting specimen was tested for mechanical properties. The mechanical properties of each group of specimens were recorded separately and finally the data were processed. The main process of the test is shown in Figure 2.

**Figure 3:**
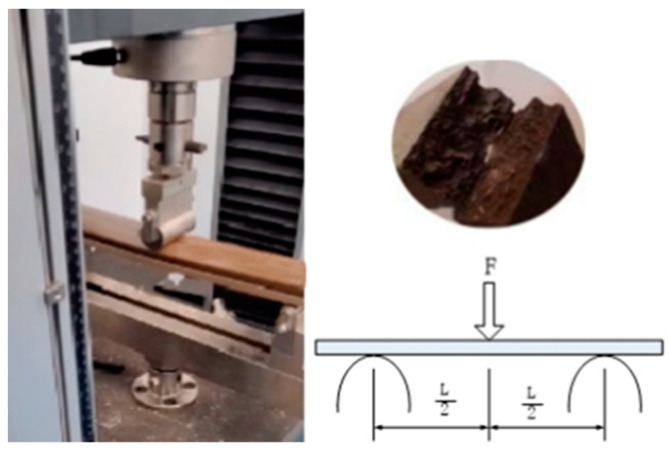
Destructive test.

The mechanical tests were carried out with reference to the ASTM D790 standard [22]. The fabricated profile was cut to the standard size required for the bending test: 164 mm × 40 mm × 4 mm. The test was carried out by placing the specimen on a GT-20A electronic universal mechanical testing machine by Xieqiang Instrument Manufacturing Co., Shanghai, China, for destructive testing. The width of the specimen B was 40 mm and the height of the specimen H was 4 mm. The span of the specimen L was 100 mm, and the speed of the loading beam was 13 mm/min. When the specimen broke, it was considered to be damaged. The test schematic is shown in Figure 3.

## 4. Result and Discussion

After the test was completed, the test results of each group of each specimen were recorded, and data processing was carried out after calculation to obtain the modulus of elasticity and bending strength for each group of specimens and recorded, and the test result data are shown in Table 2.

### 4.1. Finite Element Simulation of Wood–Plastic Composites

**Figure 4:**
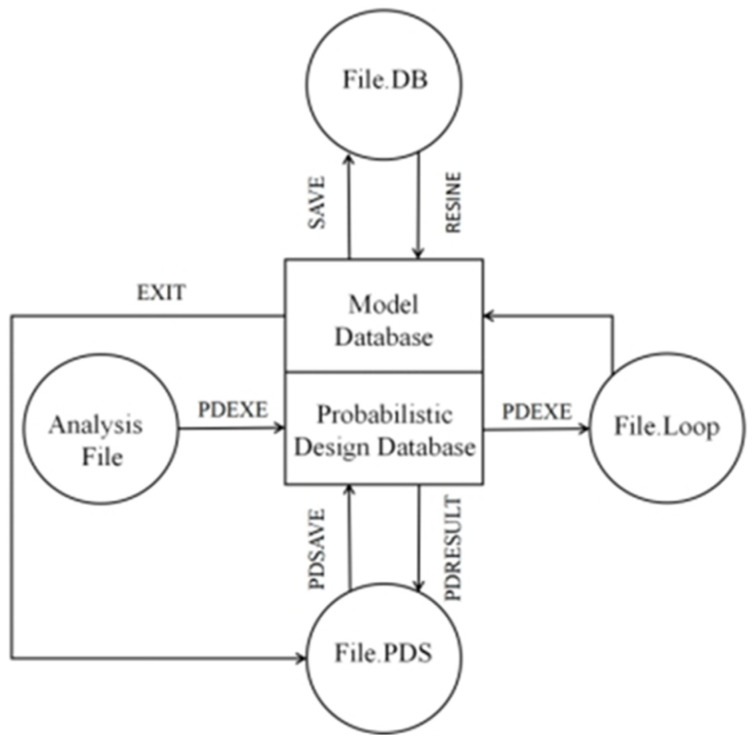
Flow chart of data processing for reliability analysis.

Finite element software has been widely used in the field of wood–plastic composites [23]. In this work, the probabilistic analysis module in the finite element software ANSYS 15.0 was used to analyze the reliability of the wood–plastic composites, and the data flow is shown in Figure 4.

This simulation test uses the second group of specimens in Table 2 as the simulation object. The specified conditions of use of the specimen are also the force conditions under the specific use environment. The reliability of wood–plastic composites as a material for building decking is studied in the construction field, where they are widely used. Because of the wide application of wood–plastic composites in the construction field, under this condition, the wood–plastic composite material can be simplified as a simple support beam model for finite element simulation. The specified service life is based on the design reference period in the relevant national standards, which is typically 50 years. The specified function is that the specimen will not be damaged in this service environment. The damage criterion for this simulation is the maximum stress theory in the mechanics of materials, i.e., the specimen is damaged when the maximum stress in the specimen reaches its bending strength. The stresses in bending can be calculated in the mechanics of materials as long as the ratio of the span of the beam to the height of the section is greater than or equal to 5. The ratio of span to section height is 25, so the effect of shear generation is not considered and only the mechanically simplified model is considered. According to the Code of Structural Loads for Buildings GB50009-2001 and the data measured in the test, span L, width B, height H and modulus of elasticity E of the specimen and the load P are taken as random variable parameters, where the load P is set to 2000 N/m.

The finite element simulation of the reliability of the wood–plastic composites is carried out according to Figure 4: the wood–plastic composites are modeled using the key points to generate the line surface, and the wood–plastic composites are reduced to isotropic linear elastomeric materials to facilitate the calculation. The input variables are defined as L, B, H, E and P, and the output variable is SMIN. The distribution type of each random variable is determined as normal distribution. The sampling analysis is performed using the Latin hypercube sampling method with 100 cycles of analysis. Then, the results of the reliability analysis are output, and the distribution graph of the values of each random variable and the probability density function are plotted. Finally, the correlation coefficient values between each input variable and the output variable SMIN are determined.

This work presents a probabilistic analysis of the reliability of wood–plastic composites. Specifically, the geometric and performance characteristics of the material and the external loads are used as random input variables to obtain a general law for the reliability of wood–plastic composites when used as building decking materials. In the following, the results of the reliability analysis are observed and analyzed.

**Figure 5:**
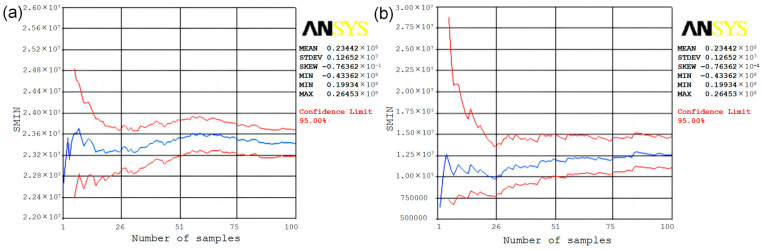
Sample of means and standard deviations of the random output variable SMIN: (**a**) mean values of samples; (**b**) standard deviation of sample.

Figure 5a shows the sample of means for the random output variable SMIN, and it can be seen from the narrow and steady convergence band in the figure that 100 cycles are sufficient. The horizontal coordinate in the figure is the number of sampling times and the vertical coordinate is the value of the maximum stress taken. It can be seen in the figure that the sample means are not symmetrical and the strength of dispersion on the right side of the data mean is significantly stronger than on the left side. The random output variable SMIN sample standard deviation is shown in Figure 5b. The horizontal coordinate is the number of sampling times and the vertical coordinate is the standard deviation. The convergence bandwidth is narrower and the standard deviation tends to smooth out.

**Figure 6:**
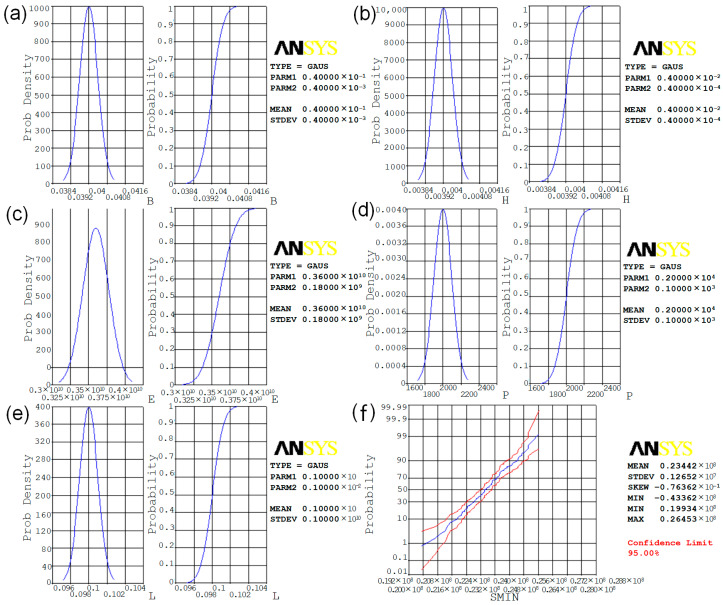
Probability distribution function for each random variable: (**a**) distribution curve of height; (**b**) distribution curve of width; (**c**) distribution curve of elastic modulus; (**d**) distribution curve of load; (**e**) distribution curve of length; (**f**) cumulative distribution function.

The range of values of each random variable and the probability distribution function are shown in Figure 6a–f. For continuous variables, the probability distribution function is also known as the probability density function. The horizontal coordinates are the values of the random variables and the vertical coordinates are the corresponding probabilities. Each random variable satisfies a normal distribution, and, from Figure 6, it can be seen that the modulus of elasticity E has the highest probability of taking a value between 3.25 × 10^9^ Pa and 3.5 × 10^9^ Pa, and the cumulative probability distribution curve reaches 1 when it reaches its maximum value. The load P, length L, width B and height H have the highest probability when taking a value near their mean values, and the cumulative probability distribution curve reaches 1 when it reaches its maximum value. The larger the maximum stress SMIN is, the closer the cumulative probability distribution curve is to 1.

**Figure 7:**
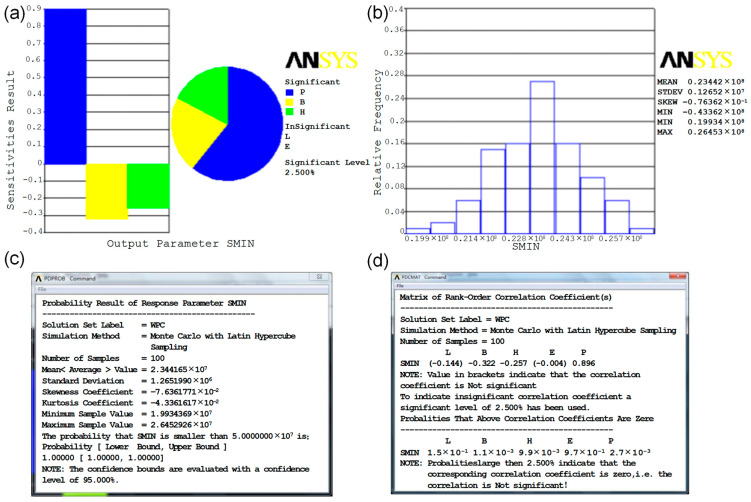
(**a**) Sensitivity of SMIN to input random variables; (**b**) histogram of maximum stress; (**c**) reliability results for specimens; (**d**) correlation coefficients of stresses with each input parameter.

The sensitivity analysis of the maximum bending stress SMIN for each input parameter is shown in Figure 7a. The threshold value of sensitivity is 2.5%. Impact levels below 2.5% are non-significant, so they can be ignored. An effect level of 2.5% or more is considered significant, and it can be seen from the figure that the uniform load P has the greatest influence on the stress, followed by the width B of the specimen, and finally the height H of the specimen. The higher the value of P, the higher the stress on the specimen, indicating that the load and the probability of failure are also higher. The negative values of the width B and height H of the specimen indicate that the greater the value, the lower the stress and, correspondingly, the lower the probability of failure. This provides a direction and theoretical guidance for the next step of the optimal design of structures. Figure 7b shows the distribution of the maximum stresses, which can be seen to be in line with the normal distribution.

Figure 7c shows the probability that the maximum stress is less than the ultimate stress of the specimen, i.e., the reliability of the material. As can be seen from Figure 7c, the reliability of the specimen under normal service conditions is 1. The minimum and maximum values of the maximum stress are 1.99 × 10^7^ Pa and 2.64 × 10^7^ Pa, respectively. The bending strength of the specimen is 5.0 × 10^7^ Pa and therefore the safety is high, with a safety factor of 2.133.

The correlation coefficients between the output variables are shown in Figure 7d. The absolute value of the correlation coefficient of load P on stress is the largest at 0.896, followed by width B at 0.322, height H at 0.2570, length L at 0.144 and finally modulus of elasticity E at 0.004. It can be seen that modulus of elasticity E and length L are in square brackets, so their coefficients are below the impact level of 2.5%, which is a non-significant impact. In the case of the significant effects, the load P is the largest and positive, indicating that its effect on the maximum stress is positive, while the width B and height H are negative, indicating that their effect on the maximum stress is negative. The positive influence on the maximum stress makes its value larger and closer to the bending strength of the test piece, i.e., the lower the reliability. The negative influence makes its value smaller and further away from the bending strength of the test piece, i.e., the higher the reliability. It can be seen that the importance of the influence on the reliability of the test piece is in the order of external load P, width B and height H. In this way, it can be considered according to the actual use when designing the structure, so that a material or system can be designed that meets the strength requirements and avoids waste.

According to the relevant knowledge of material mechanics, the force of the wood–plastic composite specimen can be simplified as the force situation of a simply supported beam. The bending moment at the midpoint cross-section of the beam is the largest, and the point at the surface of the beam is the first to be damaged. The maximum stress at the point is obtained from the bending moment calculation formula of the beam, i.e., the maximum stress of the beam is

$$\mu_{max}=\frac{M_{max}y_{max}}{I}=\frac{3PL^{2}}{4BH^{2}}=2.34\times 10^{7}\;Pa$$

$\mu_{max}$ is the maximum stress at the midpoint, $M_{max}$ is the bending moment of the cross-section at the midpoint, $y_{max}$ is the maximum distance to the neutral layer, i.e., half of the H I is the rotational inertia of the cross-section, P is the load, L is the distance between two support points, B is the width of the cross-section, and H is the height of the cross-section.

This result is consistent with the mean SMIN values obtained from the ANSYS simulations and shown in Figure 7c, so the modeling of the specimen and the application of constraints and loads are accurate. Since the convenience and accuracy of the simulations have been verified for a large number of calculations and the results obtained from the simulations are intuitive and comprehensive, the simulations will be used to simulate the 20 sets of specimens made in the following section under the same operating environment in order to investigate the differences in the reliability. The following finite element simulations will be used to investigate the differences in reliability and the intrinsic links between the different processing processes of the wood–plastic composites under the same conditions of use.

### 4.2. Simulation of the Influence of Process Parameters on Reliability

The reliability of wood–plastic composites is influenced by a number of factors, including the geometrical characteristics of the material and the external loads entered as random variables in the above section, while the process parameters of the wood–plastic composites themselves have a significant influence on their performance and thus affect the reliability of their use to varying degrees. Twenty groups of specimens are used as simulated objects, and the reliability of each group of specimens in the same operating environment is investigated in terms of the wood flour content, granulation temperature, coupling agent content and screw speed of the wood–plastic composites.

#### 4.2.1. Simulation of the Effect of Wood Flour Content on the Reliability of Wood–Plastic Composites

The wood flour content plays a decisive role in the performance of wood–plastic composites, which, in many cases, are actually used instead of wood. It is more representative to first investigate the effect of wood flour content on reliability. The process parameters for each group of simulated specimens are shown in Table 1, and the performance parameters for the specimen simulations are shown in Table 2a. The reliability calculations are carried out after the finite element simulations for the specimens with different wood flour content, respectively, and the reliability of each numbered specimen is obtained, as shown in Table 3.

From Table 3, we can see that there exists an optimum value of wood flour content around 60%. The effect of wood flour content on the reliability and safety factor is positive until it reaches the optimum value, and when the wood flour content exceeds the optimum value, the reliability remains the same and the safety factor decreases. As a whole, the safety factor increases from 0.896 to 1.387, a change of 55%.

#### 4.2.2. Simulation of the Effect of Granulation Temperature on the Reliability of Wood–Plastic Composites

The granulation temperature is a very important process parameter in the manufacture of wood–plastic composites, so it is also important to design simulations of its effect on the reliability of wood–plastic composites. As in the previous analysis, the process parameters for specimen fabrication are shown in Table 1 and the performance parameters for the reliability simulations are shown in Table 2b. Finite element simulations are carried out for different granulation temperatures of the wood–plastic composites, their effect on reliability is analyzed, and the results obtained are shown in Table 4.

From Table 4, it can be seen that the granulation temperature has a positive effect on the reliability and safety factor in the interval from 130 °C to 150 °C. The safety factor increases from 0.853 to 1.237, a change of 45%, indicating that the granulation temperature also has an effect on the reliability of the wood–plastic composites, but the effect is not as great as the effect of wood flour content on reliability.

#### 4.2.3. Simulation of the Effect of Coupling Agent Content on the Reliability of Wood–Plastic Composites

A coupling agent is a reinforcing agent, generally used in composite materials to improve their mechanical properties. Coupling agents, in the processing of wood–plastic composites, can react with both the matrix and the groups on the surface of the material. The coupling agent can form an interface layer between the reinforcing material and the matrix to transfer stress, so that the performance of the composite material is improved. Commonly used coupling agents are silanes, titanates, phosphate esters, chromium complexes, etc. This test uses maleic anhydride-grafted polyethylene, which is the most commonly used coupling agent in the production of wood–plastic composites. Before use, the coupling agent should be crushed in a grinder so that it can be fully mixed with the matrix and reinforcing materials.

The processing parameters of the wood–plastic composites with different coupling agent content are shown in Table 1, and the various performance parameters under the same simulation environment are shown in Table 2c. The finite element simulation results of the wood–plastic composites with different coupling agent content are shown in Table 5.

Through the data in Table 5, we can find that there is an optimal value of coupling agent content around 3%. When the coupling agent content does not reach the optimal value, the increase in coupling agent content has a positive effect on the reliability of wood–plastic composites. When the coupling agent content exceeds this optimal value, the reliability remains unchanged and the safety factor decreases. The safety factor increases from 1.066 to 1.493, a change of 40%, which shows that the effect of the coupling agent on the reliability of wood–plastic composites is less than the effect of the granulation temperature.

#### 4.2.4. Simulation of the Effect of Screw Speed on the Reliability of Wood–Plastic Composites

The screw speed of the extrusion molding machine is a direct factor affecting the extrusion rate of the molding machine, which indirectly affects the variation in the geometric parameters of the extruded profile and the composition structure of its internal components, so it is of some significance to study the influence of the screw speed on reliability. The process parameters for the manufacture of the designed rotational speed on reliability simulation test specimens are shown in Table 1 and the simulated performance parameters of the specimens are shown in Table 2d. The results of the screw speed on the reliability of wood–plastic composites obtained from the simulations under the same use environment are shown in Table 6.

As can be seen from the data in Table 6, in the speed range from 60 r/min to 180 r/min, the effect of speed on reliability is first negative and starts to change to a positive effect when it reaches the worst value, which is around 120 r/min. The safety factor changes from 1.130 to 1.557, a change of 37.78%, so it is concluded that the effect of screw speed on reliability is not as great as the effect of the coupling agent on reliability.

We can see from the results that there is an optimum value for the effect of wood flour content on reliability, so we should take a value close to the optimum value for wood flour content when producing wood–plastic composites in order to ensure their reliability. The influence of the granulation temperature on the reliability, with this range of values, shows an upward trend. If available, a larger granulation temperature can be selected on the basis of a combination of energy utilization and reliability. The influence of the coupling agent content on the reliability of wood–plastic composites is similar to that of the wood flour content, and a value close to the optimum value of the coupling agent content should be taken to ensure the reliability of the material. The influence of the screw speed on the reliability of wood–plastic composites is unique, and, from the test results, there is a minimal value for its influence on the reliability. Therefore, in order to ensure the reliability of the material, it is important to avoid the minimal value as far as possible.

## 5. Conclusions

In this work, the reliability of test pieces in the same use environment is simulated according to the difference in manufacturing process parameters. The reliability of test pieces under different processing parameters and their safety factors are obtained. The importance of the influence of each processing parameter on reliability is discussed through horizontal comparison. The results show the following.

(1) The geometric and mechanical property parameters of the specimen and the external load that affect the reliability are taken as the basic random variables, and the maximum stress damage criterion is applied as the limit state equation. The reliability of the specimen is analyzed using the probabilistic analysis module of the finite element simulation software ANSYS. The absolute values of the correlation coefficients between the maximum bending stress and the loads P, B, H, L and E are 0.896, 0.322, 0.2570, 0.144 and 0.004, respectively. From the results of the analysis, it is found that, in addition to the load, the greatest influence on the reliability of the specimen is the width and height of the specimen when the wood–plastic composite material is used as a building decking material.

(2) In this work, the reliability of each group of specimens prepared under different processing parameters is simulated under the same environment, and the degree of influence of each process parameter on the reliability is obtained by the change in safety coefficient: the influence of wood flour content was the largest, with a change of 55%, followed by the granulation temperature with a change of 45%, the content of coupling agent with a change of 40% and the screw speed with a change of 37.78%.

The research in this work provides a certain reference basis for the optimization of wood–plastic composites’ manufacturing process parameters. The reliability analysis methods and results used in this work can also be simulated and optimized for the improvement of wood–plastic composites in other structures to avoid safety accidents and economic losses.

In this work, we only analyze the reliability problems when they are used as building paving materials. In the subsequent research, we will cover as much as possible the use fields of wood–plastic composites to obtain more statistical data to guide the processing of wood–plastic composites.

## Figures and Tables

**Table 1:** Process parameters for each group of specimens required for the test.

Specimen Group	Wood Flour Content	Granulation Temperature (°C)	Coupling Agent Content	Screw Speed (r/min)
1~5	30, 40, 50, 60, 70	140	3	120
6~10	30	130, 135, 140, 145, 150	3	120
11~15	40	140	1, 2, 3, 4, 5	120
16~20	50	140	3	60, 90, 120, 150, 180

**Table 2:** Simulation performance parameters for the effect of each variable on reliability.

(a) The effect of wood flour content on reliability				
**Specimen Number**	**Wood Flour Content** **/%**	**Modulus of Elasticity/Pa**		**Bending Strength** **/Pa**
		**Average Value**	**Standard Deviation**	
1	30	2.2 × 10^9^	1.1 × 10^8^	4.2 × 10^7^
2	40	3.6 × 10^9^	1.8 × 10^8^	5.0 × 10^7^
3	50	4.9 × 10^9^	2.5 × 10^8^	5.6 × 10^7^
4	60	4.8 × 10^9^	2.4 × 10^8^	6.5 × 10^7^
5	70	4.4 × 10^9^	2.2 × 10^8^	6.0 × 10^7^
Distribution Type	-	Gaussian distribution		-
(b) The effect of granulation temperature on reliability				
**Specimen Number**	**Granulation Temperature/** **°C**	**Modulus of Elasticity/Pa**		**Bending Strength** **/Pa**
		**Average Value**	**Standard Deviation**	
6	130	2.0 × 10^9^	1.0 × 10^8^	4.0 × 10^7^
7	135	2.5 × 10^9^	1.25 × 10^8^	5.5 × 10^7^
8	140	3.0 × 10^9^	1.5 × 10^8^	5.2 × 10^7^
9	145	2.4 × 10^9^	1.2 × 10^8^	5.3 × 10^7^
10	150	2.8 × 10^9^	1.4 × 10^8^	5.8 × 10^7^
Distribution Type	-	Gaussian distribution		-
(c) The effect of coupling agent content on reliability				
**Specimen Number**	**Screw Speed** **r/min**	**Modulus of Elasticity/Pa**		**Bending Strength** **/Pa**
		**Average Value**	**Standard Deviation**	
16	60	5.0 × 10^9^	2.5 × 10^8^	6.8 × 10^7^
17	90	4.9 × 10^9^	2.45 × 10^8^	5.7 × 10^7^
18	120	4.2 × 10^9^	2.1 × 10^8^	5.3 × 10^7^
19	150	5.0 × 10^9^	2.5 × 10^8^	7.2 × 10^7^
20	180	4.9 × 10^9^	2.45 × 10^8^	7.3 × 10^7^
Distribution Type	-	Gaussian distribution		-
(d) The effect of rotation speed on reliability				
**Specimen Number**	**Coupling Agent Content** **%**	**Modulus of Elasticity/Pa**		**Bending Strength** **/Pa**
		**Average Value**	**Standard Deviation**	
11	1	3.4 × 10^9^	1.7 × 10^8^	5.0 × 10^7^
12	2	3.6 × 10^9^	1.8 × 10^8^	5.0 × 10^7^
13	3	4.0 × 10^9^	2.0 × 10^8^	7.0 × 10^7^
14	4	3.8 × 10^9^	1.9 × 10^8^	6.7 × 10^7^
15	5	3.6 × 10^9^	1.8 × 10^8^	6.5 × 10^7^
Distribution Type	-	Gaussian distribution		-

**Table 3:** Results of the wood flour content on reliability.

Specimen Number	Wood Flour Content/%	Maximum Stress Mean Value/Pa	Bending Strength/Pa	Reliability/%	Safety Factor
1	30	4.688 × 10^7^	4.2 × 10^7^	2.971	0.896
2	40	4.688 × 10^7^	5.0 × 10^7^	88.373	1.067
3	50	4.688 × 10^7^	5.6 × 10^7^	100	1.195
4	60	4.688 × 10^7^	6.5 × 10^7^	100	1.387
5	70	4.688 × 10^7^	6.0 × 10^7^	100	1.280

**Table 4:** Simulation results of the granulation temperature on reliability.

Specimen Number	Granulation Temperature/°C	Maximum Stress Mean Value/Pa	Bending Strength/Pa	Reliability/%	Safety Factor
6	130	4.690 × 10^7^	4.0 × 10^7^	0	0.853
7	135	4.690 × 10^7^	5.2 × 10^7^	96.890	1.109
8	140	4.690 × 10^7^	5.3 × 10^7^	98.480	1.130
9	145	4.690 × 10^7^	5.5 × 10^7^	99.059	1.173
10	150	4.690 × 10^7^	5.8 × 10^7^	100	1.237

**Table 5:** Simulation results of the coupling agent content on the reliability of WPC.

Specimen Number	Coupling Agent Content %	Maximum Stress Mean/Pa	Bending Strength/Pa	Reliability/%	Safety Factor
11	1	4.690 × 10^7^	5.0 × 10^7^	92.362	1.066
12	2	4.690 × 10^7^	5.0 × 10^7^	92.362	1.066
13	3	4.690 × 10^7^	5.3 × 10^7^	100	1.493
14	4	4.690 × 10^7^	5.5 × 10^7^	100	1.429
15	5	4.690 × 10^7^	5.8 × 10^7^	100	1.386

**Table 6:** Simulation results of the screw speed on reliability.

Specimen Number	Screw Speed/r/min	Maximum Stress Mean Value/Pa	Bending Strength/Pa	Reliability/%	Safety Factor
16	60	4.690 × 10^7^	6.8 × 10^7^	100	1.450
17	90	4.690 × 10^7^	5.7 × 10^7^	100	1.215
18	120	4.690 × 10^7^	5.3 × 10^7^	98.480	1.130
19	150	4.690 × 10^7^	7.2 × 10^7^	100	1.535
20	180	4.690 × 10^7^	7.3 × 10^7^	100	1.557

## Data Availability

Not applicable.

## References

[1] Sobczak L., Brüggemann O., Putz R.F. (2012). Polyolefin composites with natural fibers and wood-modification of the fiber/filler–matrix interaction. J. Appl. Polym. Sci..

[2] Sobczak L., Lang R.W., Haider A. (2012). Polypropylene composites with natural fibers and wood – General mechanical property profiles. Compos. Sci. Technol..

[3] Alrubaie M.A.A., Lopez-Anido R.A., Gardner D.J., Tajvidi M., Han Y. (2019). Experimental investigation of the hygrothermal creep strain of wood–plastic composite lumber made from thermally modifified wood. J. Thermoplast. Compos. Mater..

[4] Alrubaie MA A., Lopez-Anido R.A., Gardner D.J., Tajvidi M., Han Y. (2019). Modeling the hygrothermal creep behavior of wood plastic composite (WPC) lumber made from thermally modified wood. J. Thermoplast. Compos. Mater..

[5] Daniel A.V. (2010). Structural Performance of Wood Plastic Composite Sheet Piling. J. Mater. Civ. Eng..

[6] Gardner D., Han Y. Towards Structural Wood-Plastic Composites: Technical Innovations. Proceedings of the 6th Meeting of the Nordic-Baltic Network in Wood Material Science and Engineering (WSE).

[7] Haiar K.J. (2000). Performance and Design of Prototype Wood-Plastic Composite Sections.

[8] Hamel S.E. (2011). Modeling the Time-Dependent Flexural Response of Wood-Plastic Composite Materials.

[9] Dan M.F., Recek S. (2003). Reliability of fiber-reinforced composite laminate plates. Probabilistic Eng. Mech..

[10] Yang H.S., Qiao P., Wolcott M.P. (2010). Fatigue characterization and reliability analysis of wood flour filled polypropylene composites. Polym. Compos..

[11] Lin S.C. (2000). Reliability predictions of laminated composite plates with random system parameters. Probabilistic Eng. Mech..

[12] Yu G.W. (2013). Nondestructive Testing and Reliability Analysis of Wood Plastic Plate.

[13] Yu G.W., Hu Y.C., Gu J.Y. (2011). Relativity Analysis between Dynamic and Static Modulus of Elasticity on Different Thickness Wood-Plastic Structural Plates. Appl. Mech. Mater..

[14] Chen N.Z., Sun H.H., Soares C.G. (2003). Reliability analysis of a ship hull in composite material. Compos. Struct..

[15] Chen N.Z., Soares C.G. (2007). Reliability assessment of post-buckling compressive strength of laminated composite plates and stiffened panels under axial compression. Int. J. Solids Struct..

[16] Slaughter A.E. (2004). Design and Fatigue of a Structural Wood-Plastic Composite.

[17] Jiang L., Fu J., He C. (2020). Reliability analysis of wood-plastic composites in simulated seawater conditions: Effect of iron oxide pigments. J. Build. Eng..

[18] Jiang L., Fu J., Liu L. (2020). Seawater Degradation Resistance of Straw Fiber-reinforced Polyvinyl Chloride Composites. BioResources.

[19] Schirp A., Plinke B., Napolow D. (2015). Effectiveness of organic and inorganic pigments for mass colouration of thermo-mechanical pulp used in wood–plastic composites. Eur. J. Wood Wood Prod..

[20] Haque M.M.U., Goda K., Ogoe S., Sunaga Y. (2019). Fatigue analysis and fatigue reliability of polypropylene/wood flour composites. Adv. Ind. Eng. Polym. Res..

[21] Yang H.S., Qiao P., Wolcott M.P. (2010). Flexural Fatigue and Reliability Analysis of Wood Flour/High-density Polyethylene Composites. J. Reinf. Plast. Compos..

[22] (2010). Standard Test Methods for Flexural Properties of Unreinforced and Reinforced Plastics and Electrical Insulating Materials.

[23] Wilczyński K., Buziak K., Wilczyński K.J., Lewandowski A., Nastaj A. (2018). Computer Modeling for Single-Screw Extrusion of Wood–Plastic Composites. Polymers.

